# Factors associated with overweight cats successfully completing a diet-based weight loss programme: an observational study

**DOI:** 10.1186/s12917-018-1740-5

**Published:** 2018-12-14

**Authors:** Erin M. O’Connell, Maria Williams, Shelley L. Holden, Vincent Biourge, Alexander J. German

**Affiliations:** 1 Institute of Veterinary Science, University of Liverpool, Neston, UK; 2 Institute of Ageing and Chronic Disease, University of Liverpool, Leahurst Campus, Chester High Road, Neston, CH64 7TE UK; 3 Royal Canin Research Center, Aimargues, France; 4 Present address: Crown Pet Foods Ltd., Oak Tree Meadow, Blackworthy Road, Castle Cary, Somerset BA7 7PH UK

**Keywords:** Feline, Overweight, Obese, Weight loss, Caloric restriction, Outcomes

## Abstract

**Background:**

The most common approach for controlled weight loss in cats is dietary caloric restriction, using a purpose-formulated diet. Most previous studies have only assessed short-term outcomes, and no previous study has examined overall success (i.e. odds of reaching target weight). The aim of this study was to determine the factors associated with overweight cats successfully completing a diet-based weight loss programme to reach target weight.

**Results:**

Sixty-two cats were included, and 28 (45%) completed their weight loss programme. The remaining 34 cats (55%) did not reach target weight, of which 2 (3%) were euthanised for unrelated reasons. Reasons for cats stopping the programme prematurely included inability to contact owner (*n* = 19), owner requested that the programme be completed prior to reaching target weight (*n* = 5), the cat developed another illness (*n* = 3), refusal to comply with requirements for weight management (*n* = 2), owner illness (*n* = 2), and personal issues of the owner (*n* = 1). Multiple logistic regression analysis revealed that rate of weight loss and weight loss required were positively (odds ratio [OR] 157.81, 95% confidence interval [CI] 10.00–2492.67) and negatively (OR 0.89, 95% CI 0.81–0.98) associated with the odds of completing the weight loss programme, respectively.

**Conclusions:**

Future studies should consider developing better methods of supporting the owners of the most obese cats during weight management, since these cats are least likely to complete reach target weight.

**Electronic supplementary material:**

The online version of this article (10.1186/s12917-018-1740-5) contains supplementary material, which is available to authorized users.

## Background

Obesity is defined as “a disease in which excessive bodyweight has accumulated to the point where health is adversely affected” [1]. In cats, this disorder is associated with various comorbidities including diabetes mellitus, gastrointestinal disease, hepatic lipidosis, hypertension, musculoskeletal disease, neoplasia, ophthalmological diseases, oral conditions, skin diseases, and both upper and lower urinary tract conditions [2–4]. Further, cats with a body condition score (BCS) of 9/9 have a shorter average lifespan than cats in ideal weight [5]. Given that recent studies suggest 16–63% of cats in the westernised world are overweight or have obesity [6–10], it is not surprising that many consider it to be the second most common health problem of cats living in developed countries [8].

Controlled weight loss in cats typically involves caloric restriction, using a purpose-formulated diet, coupled with increased activity [11, 12]. Several previous studies have shown positive results for such an approach, although most studies have involved colony cats with experimentally-induced obesity, rather than client-owned cats with naturally occurring disease [11–21]. Many of these studies have examined the effect of various factors on weight loss, including diet type and composition, and the weight loss outcomes assessed have included rate of weight loss, percentage of weight lost, and changes in body composition. For example, increasing dietary protein content minimises the loss of lean tissue but does not alter either rate or percentage of weight lost [14, 15]. Further, although there is no difference in percentage of weight lost when either dry or wet therapeutic weight loss diets are used [18], one study suggested that a lower daily energy intake was needed to maintain the same rate of weight loss when cats that were fed either wet food or a combination of wet and dry food [17]. In another study, despite no difference in average percentage of weight lost, overweight cats fed dry food exclusively during a short-term (12-week) weight loss programme, were more likely to complete than cats fed a mix of wet and dry food [21].

Other variables assessed in feline weight loss studies have included sex and neuter status, neither of which appear to influence either the rate or percentage of weight lost [17, 18, 21]. However, this contrasts with a recent study in overweight dogs where female dogs lost more weight than male dogs [22]. Further, although age has not been associated with differences in outcome of the weight loss phase [17, 18, 21], a recent study revealed that cats over 9y were less to regain weight after successfully reaching their target [23]. Finally, environment might also influence success, not least since different outcomes have been observed according to geographical location [21].

Whilst previous studies have provided useful insights into factors that might influence weight loss outcomes, the main limitation most of these studies have only assessed short periods of weight loss (i.e. from the first 2–6 months) and have not evaluated the success of weight loss programme as a whole (i.e. whether target weight is actually reached). Such an outcome was examined in a recent weight loss study in overweight dogs, and just half of the dogs completed successfully [24]. The odds of a dog successfully reaching target weight were positively associated with feeding a dry therapeutic diet, the overall time taken to reach target, and the rate of weight loss, but negatively associated with starting body fat percentage, and use of the weight-loss drug, dirlotapide. A further long-term weight loss study in dogs revealed associations between outcomes and the presence of concurrent disease [25]. In light of the fact that such a study has not yet been undertaken in cats, our aims were to determine the proportion of cats that successfully completed a diet-based weight loss programme to reach target weight, and to determine factors associated with success.

## Results

### Study animals and outcomes of weight loss

During the study period 79 cats were referred to the RCWMC. Of these, 62 met the inclusion criteria i.e. there was a known outcome for their weight loss. Of those not included, no weight loss was deemed to be necessary in six cats, while the remaining eleven were not eligible because the weight loss period was still ongoing at the time of data review. The study population comprised 38 male cats (37 neutered, one entire) and 24 female neutered cats. The median age at enrolment was 90 months (7.7 years), with a range of 16 months (1.3 years) to 156 months (13.0 years). Breeds included were 54 domestic shorthair cats, one domestic longhair cats and 7 pedigree cats (2 British blue, 1 Maine coon, 1 Selkirk rex and 2 Siamese). Forty-five cats (73%) had at least one concurrent disease, including orthopaedic (10), cardiorespiratory (*n* = 7), gastrointestinal (*n* = 21), urinary (*n* = 7), oral/dental (*n* = 9), dermatological (*n* = 12) and other (*n* = 5) conditions.

Full details of all cats included in the study are given in Table 1, whilst details of the outcomes of weight loss are given in Table 2. Of the 62 cats, 28 (45%) completed their programme to reach their target weight, 32 (52%) stopped prematurely, failing to reach their target weight, and 2 (3%) were euthanised at the owners’ request. One cat was euthanised as a result of spinal neoplasia, whereas the second was euthanised due to perceived poor quality of life. The reasons for the 32 cats stopping the programme prematurely included inability to contact the owner (*n* = 19), the owner requested the programme be completed prior to reaching target weight (*n* = 5), the cat developed another illness (*n* = 3), refusal to comply with requirements for weight management (*n* = 2), owner illness (*n* = 2), and personal issues of the owner (*n* = 1). Median duration of the weight loss phase was 177 days (range 40 to 796 days) and 159 days (range 1 to 758 days), respectively, for the cats that completed and stopped early. Further, in the cats that completed lost a median of 18% SBW (range 6 to 37.4%) and median rate of weight loss was 0.7% per week (range − 0.2% per week to 3.1% per week); by contrast, for cats that stopped early, median percentage weight loss was 8% SBW (range − 2 to 16%) and median rate of weight loss was 0.2% per week (range − 0.2% per week to 0.9% per week). The rate of weight loss in both cats that died was 0.3% per week, with one cat losing 12% of starting body weight over 251 days, and the second losing 6% over 119 days.

**Table 1:** Baseline parameters of the cats in the study population

Parameter	Completed (*n* = 28)	Stopped (*n* = 32)	Died (*n* = 2)
Breed^a^	DSH 24	DSH 29	DSH 1
	British Blue 2	British Blue 0	British Blue 0
	Maine Coon 0	Maine Coon 1	Maine Coon 0
	Siamese 1	Siamese 0	Siamese 1
	Selkirk Rex 0	Selkirk Rex 1	Selkirk Rex 0
	BSH 1	BSH 0	BSH 0
	DLH 0	DLH 1	DLH 0
Sex	MN 17, FS 11	MN 18, FS 13	MN 2, FS 0
	M 0, F 0	M 1, F 0	M 0, F 0
Age (months)	95 (24–156)	84 (16–156)	150 & 113
Lean body mass (kg)^a^	4.0 (2.9–5.5)	4.0 (2.9–5.7)	4.1 & 5.6
Body fat (%)^a^	30 (17–45)	35 (24–50)	30 & 54
Weight loss required (%)^b^	18 (6–37)	28 (9–53)	19 & 62
Concurrent disease	Present 21	Present 22	Present 3 (2 & 1)
	Absent 7	Absent 10	Absent 0

All data are expressed as the median (range) unless otherwise stated *BSH* British Short Hair, *DSH* Domestic Shorthair, *DLH* Domestic Longhair, *F* female, *FS* female spayed, *M* male, *MN* male neutered. ^a^Lean body mass and body fat percentage determined before weight loss by dual-energy x-ray absorptiometry (DEXA). ^b^Weight loss required calculated from DEXA results using a mathematical formula based upon typical body composition results from previous weight clinic studies [17, 23]

**Table 2:** Comparison of the results of weight loss amongst groups

Parameter	Completed (*n* = 28)	Stopped (*n* = 32)	Died (*n* = 2)
Diet^a^	Mixed 15	Mixed 8	Mixed 1
	HPHF dry 7	HPHF dry 8	HPHF dry 0
	HPMF dry 6	HPMF dry 15	HPMF dry 1
	HPMF wet 0	HPMF wet 1	HPMF wet 0
Diet type changed	11	25	0
Energy intake^a^	31 (18 to 39)	34 (22 to 51)	25 & 59
Weight loss stalls^b^	2 (0 to 14)	2 (0 to 8)	2 & 0
Dietary energy reductions^c^	1 (0 to 13)	2 (0 to 11)	2 & 1
Actual weight lost (%)^d^	18 (6 to 37)	8 (−2 to 16)	12 & 6
Rate of weight loss^e^	0.7 (0.3–3.1)	0.2 (−0.2 to 0.9)	Both 0.3
Duration (d)	177 (40–796)	159 (1–758)	251 & 119

For cats that either completed or stopped, data are expressed as median (range). Given that only two cats died, individual results are instead reported, with the same order used for results from each cat. HPHF: High protein high fibre (Royal Canin Satiety support); HPMF: High protein medium fibre (Royal Canin Obesity support). ^a^Average energy intake calculated as kcal per kg of ideal bodyweight. ^b^Weight loss stalls are the number of times where the patient failed to lose weight between clinic visits. ^c^Dietary energy reductions are the number of times the daily energy intake had to be reduced due to slow progress. ^d^Actual weight lost are expressed as a percentage of starting body weight. ^e^Rate of weight loss was calculated as percentage of starting body weight lost per week

### Logistic regression analysis to determine factors associated with success

First, cats that completed their weight loss programme were compared with those that stopped or died (combined). Using simple logistic regression (Table 3), rate of weight loss (OR 262.09, 95% CI 15.91–4317.10) and actual weight lost (OR 1.15, 95% CI 1.06–1.23) were positively associated with odds of completing, while starting body fat percentage (OR 0.90, 95% CI 0.83–0.98), weight loss required (OR 0.89, 95% CI 0.82–0.96), and average energy intake (OR 0.89, 95% CI 0.80–0.99) were negatively associated with odds of completing. No other factors qualified for inclusion (*P* < 0.1) in multiple logistic regression modelling.

**Table 3:** Simple logistic regression results determining factors associated with cats completing a weight loss programme

Logistic regression	Completed vs. stopped or died^a^			Completed vs. stopped only^b^		
	OR^c^	95% CI^d^	*P* value	OR^c^	95% CI^d^	*P* value
Age	1.00	0.99–1.02	0.722	1.00	0.99–1.02	0.538
*Sex*						
Female	Ref			Ref		
Male	0.96	0.34–2.67	0.933	1.06	0.375–2.98	0.916
Breed						
Mixed breed	Ref			Ref		
Pedigree	1.72	0.35–8.44	0.502	2.50	0.42–14.83	0.313
Lean mass	1.00	0.99–1.00	0.689	1.00	0.99–1.00	0.889
Body fat (%)	**0.90**	**0.83–0.98**	**0.012**	**0.90**	**0.83–0.98**	**0.015**
Concurrent disease						
Present vs absent	1.25	0.40–3.87	0.699	1.36	0.43–4.24	0.593
> 2 concurrent diseases	**0.21**	**0.02–1.96**	**0.172**	**0.20**	**0.02–1.83**	**0.154**
Orthopaedic	0.97	0.23–4.00	0.962	0.90	0.22–3.74	0.885
Dermatological	0.52	0.15–1.76	0.294	0.65	0.18–2.29	0.504
Alimentary	1.33	0.46–3.88	0.598	1.42	0.48–4.23	0.529
Cardiovascular	1.25	0.28–5.52	0.768	1.17	0.26–5.17	0.839
Urinary	2.67	0.45–15.78	0.280	2.50	0.42–14.83	0.313
Oral	**3.48**	**0.62–19.52**	**0.157**	**3.26**	**0.58–18.35**	**0.180**
Diet type						
Dry	Ref			Ref		
Wet of mixed food	**2.02**	**0.73–5.64**	**0.177**	**2.31**	**0.82–6.56**	**0.114**
Diet macronutrients						
HPHF	Ref			Ref		
HPMF	0.91	0.33–2.49	0.856	0.90	0.32–2.49	0.835
Weight loss stalls	1.07	0.87–1.31	0.542	1.05	0.86–1.29	0.630
Dietary energy reductions	0.93	0.75–1.14	0.473	0.92	0.74–1.13	0.427
Switched diet type	2.50	0.81–7.69	0.111	2.31	0.75–7.16	0.146
Weight loss required	**0.89**	**0.82–0.96**	**0.002**	**0.88**	**0.82–0.96**	**0.002**
Actual weight lost	**1.15**	**1.06–1.23**	**< 0.001**	**1.14**	**1.06–1.23**	**< 0.001**
Energy intake	**0.89**	**0.80–0.99**	**0.041**	**0.88**	**0.78–0.99**	**0.040**
Duration of weight loss	1.00	0.99–1.00	0.528	1.00	0.99–1.00	0.564
Rate of weight loss	**262.09**	**15.91–4317.10**	**< 0.001**	**221.70**	**14.02–3504.93**	**< 0.001**

^a^Cats that completed compared with cats that had either stopped or died; ^b^Cats that completed compared with cats that stopped only (i.e. those that died were excluded); ^c^OR: odds ratio; ^d^95% CI: 95 % confidence interval: Reference category used in logistic regression. For an explanation of the variables used, please see Tables 1 and 2. Variables highlighted in bold qualified for inclusion in the multiple regression analysis at *P* < 0.1 (Table 4)

The initial multiple logistic regression model included the four variables described above and, after refining by backwards stepwise elimination, the best-fit model included only two factors: rate of weight loss and weight loss required (Table 4). Rate of weight loss was positively associated with completing a weight loss programme (OR 157.81, 95% CI 10.00–1292.67), meaning that cats that lost weight more rapidly were more likely to reach target weight. In contrast, weight loss required was negatively associated with the odds of completing (OR 0.89, 95% CI 0.81–0.98), meaning that cats that needed to lose the most weight were least likely to reach target weight. When data were reanalysed by comparing cats that completed with those that stopped (i.e. after excluding cats that died), the results were unaltered (Tables 3 and 4).

**Table 4:** Multiple logistic regression results determining factors associated with cats completing a weight loss programme

Logistic regression	Completed vs. stopped & died^a^			Completed vs. stopped only^b^		
	OR^c^	95% CI^d^	*P* value	OR^c^	95% CI^d^	*P* value
Weight loss required	0.89	0.81–0.98	0.022	0.89	0.80–0.98	0.020
Rate of weight loss	157.81	10.00–2492.67	< 0.001	142.28	9.23–2192.68	< 0.001

^a^Cats that completed their weight loss programme compared with cats that had either stopped or died; ^b^Cats that completed their weight loss programme compared with cats that stopped only (i.e. those that died were excluded); ^c^OR: odds ratio; ^d^95% CI: 95 % confidence interval. For an explanation of the variables used, please see Tables 1 and 2

## Discussion

To the authors’ knowledge, this is the first study to examine the odds of obese client-owned cats reaching target weight on a weight management programme and to determine the factors associated with overall success. Unlike most previous weight loss studies, cats were followed until their period of weight loss ended, either because the cats reached target weight, or their programme ended prematurely. Not surprisingly, therefore, the duration of the weight loss reported in this study is much longer than that reported in most previous studies [11–21]. Approximately half of the cats enrolled successfully reached their target body weight, confirming the results of earlier studies on the use of weight management programmes in obese pet cats [17, 18, 23]. Nonetheless, since the remaining cats stopped their weight programme prematurely, reaching target weight is a challenging goal for many obese cats, as reported for dogs [24]. The proportion of cats completing this weight loss regimen is less than previously been reported in studies of weight loss in colony cats with experimentally-induced obesity in which the vast majority complete their programme [11–16, 18, 20]. This is not surprising since food intake can be better controlled in a colony setting, compared with pet cats undergoing weight loss supervised by their owner where compliance is a major concern [17]. This, coupled with the findings from a recent study suggesting that approximately half of all cats that reach their ideal weight subsequently regain weight [23], suggests that long-term success can be hard to achieve despite best intentions. That said, although many cats did not reach their target weight, many of them still managed to lose some weight, with the average weight loss in the cats not completing being 8% of starting body weight. One study in obese dogs with osteoarthritis suggested that weight loss of approximately 6–9% is sufficient to lead to measurable benefits in mobility, measured by force plate [26]. Although, to the authors’ knowledge, no similar studies have yet been conducted in cats, it is plausible to assume similar benefits might exist. Therefore, being unsuccessful in reaching ideal weight does not necessarily mean that the weight loss programme was without any benefit. In the authors’ opinion, rather than focusing on parameters such as percentage weight loss, rate of weight loss, or reaching target weight, it would be preferable instead to focus more on improved health metrics (i.e. decreased severity of concurrent disease, improved insulin sensitivity) and quality of life [27].

The two main factors associated with overall success in this study were rate of weight loss and the weight loss required to reach target weight. Rate of weight loss was strongly and positively associated with the odds of achieving target weight, although the confidence intervals for this variable were wide (ranging from 10 to 2493), making it difficult to be certain as to the true odds of this association. Further, given the observational nature of the study, the reason for this association is not clear. However, one explanation might be that owners who observe rapid results in terms of weight loss will be more motivated to continue the plan long-term. Alternatively, a slow rate of weight loss might be a proxy measure for poor owner compliance with the weight loss programme. Of course, additional work would be required to explore the significance of this result further. In contrast to rate of weight loss, the weight loss required to reach target body weight was negatively associated with achieving target weight meaning that the most overweight cats were least likely to reach target body weight. This finding reflects previous studies in dogs [27, 28] and people [29] where weight loss tends to plateau over time. One possible explanation might be that, when faced with a challenging target for weight loss, owners are dissuaded from continuing. Alternative explanations could include the fact that weight loss slows and becomes more challenging over time, making compliance more difficult, or the possibility that magnitude of overweight at the outset predicts clients that are more likely to be non-compliant (i.e. by feeding extra food). Ideally, prospective studies of the cats’ and owners’ lifestyle and behaviour would be required to identify the cause for this association. Indeed, a close owner-cat relationship was previously identified as a risk factor for obesity in cats [30].

A range of dietary factors were examined during the study including diet type (both in terms of macronutrient content and formulation), whether the diet type was changed, the average energy intake during the programme, and the number of times energy intake had to be reduced. None of these factors were associated with overall success. The finding that there was no association between diet type and the odds of reaching target weight contrasts with a recent study in dogs, whereby those fed a dry weight loss diet were most successful [24], and also with earlier work in cats whereby a greater level of energy restriction was required for cats either fed a wet diet or a mixture of wet and dry compared with cats fed dry food exclusively [17]. Further, whilst recent studies suggested that consumption of dry food was a risk factor for feline obesity [10, 31], and that feeding a moist diet and thereby increasing water intake contributed to higher activity levels [32, 33], the evidence from this paper does not suggest that diet type affects the chances of a cat completing a weight loss programme. The fact that dietary alterations were not associated with success suggests that making adaptations to an individual cat’s weight loss programme does not harm the process and that clients were not discouraged from continuing the weight loss programme despite a stall in weight loss.

One of the main limitations of this study was the retrospective observational design. Whilst intriguing associations were found with success of weight loss, causality cannot be assumed, and further investigations are now required to better clarify the reasons for success and failure in individual cases. A second important limitation was the use of a small referral population meaning that results from the study might not be generalisable to obese cats treated in primary care practice. For example, by selecting owners who were willing to be referred to a specialist weight management service, we might have inadvertently selected for owners who were more motivated and thus more likely to achieve success. Conversely, it might be argued that we selected for cats less likely to be successful, since several of the cats were referred following initial failure to lose weight at their primary care practice. Third, although a sample size calculation was performed, and approximately twice the required number of cats were recruited, this was based on data from dogs [24] and only one variable (percentage weight loss) was used. As a result, we cannot be certain that the study was adequately powered for all variables considered, and thus cannot exclude the possibility type II error, not least for factors with minor effects on the odds of ran obese cat reaching target weight might have been missed. In the future, larger scale, multicentre, weight loss studies should be conducted, incorporating primary care practices from diverse geographical locations. A final limitation was the fact that physical activity was not objectively measured during the study and, as a result, the influence it might have played on weight loss in cats is not clear. In humans, exercise can help to prevent the decrease in energy expenditure during weight loss [34, 35], whereas studies from dogs suggest that it might help to preserve lean tissue mass [36]. Further, one previous study suggested that environmental enrichment and increased physical activity can benefit overweight cats and their owners [37]. Physical activity was also not objectively measured in a recent canine study with a similar design to the current study, whereby 61% reached their target weight [24]. It is plausible that an increased ability to exercise dogs could have contributed, in part, to increased success in achieving target body weight in dogs compared to cats. This is speculative and without the available data an association cannot be made.

## Conclusion

The current study has demonstrated that approximately half of cats enrolled in a weight loss programme reach their target weight. While the most obese cats are less likely to succeed, owners should not be discouraged from enrolling their cat in a weight loss programme based on signalment or concurrent disease status.

## Methods

### Animals

This retrospective observational study comprised client-owned cats referred to the Royal Canin Weight Management Clinic (RCWMC), University of Liverpool, United Kingdom, for management of obesity. All cases were recruited, investigated, and managed by the same veterinarian (AJG) and registered veterinary nurse (SLH). The approach taken in this study was similar to that for a recent study in dogs [24], with cats being eligible for inclusion if their weight loss programme had commenced between December 2004 and December 2012, and whose weight loss had ended by July 2013 when all data were reviewed. In this regard, weight loss was deemed to have ended either if the cat had completed their programme reaching target weight, had stopped their programme before reaching target weight, or had died (including those that were euthanised) prior to having completed their programme. Cats whose weight loss programme was still ongoing in July 2013 were not eligible for inclusion. Further, cats had to be suitable candidates for weight loss (e.g. systemically well and with no significant abnormalities on haematology, serum biochemistry and urinalysis) and weight loss needed to be necessary (e.g. cats had a body condition score of > 5/9). The age of the cat was not considered to be a reason for exclusion. The study was performed in adherence to the University of Liverpool Animal Ethics Guidelines and the study protocol was approved by the University of Liverpool Research Ethics Committee, the Waltham ethical review committee, and the Royal Canin Ethics Committee. The owners of all participating cats gave informed written consent.

### Weight loss regimen

Details of the weight loss regimen used at the RCWMC have been previously described [17, 23]. Briefly, at the initial consultation physical examination was performed and body weight was measured with electronic weigh scales that were regularly calibrated using certified test weights. In order to ensure that cats were systemically well, routine haematology, serum biochemistry, total thyroxine measurement and urinalysis were performed. A body condition score was also assigned from a nine-integer scale [38] and body composition was analysed by fan-beam duel energy x-ray absorptiometry (DEXA; Lunar Prodigy Advance; GE Lunar) [17, 23]. The body composition results were used to estimate ideal weight: briefly, lean mass, fat mass and bone mineral content results were entered into a computer spreadsheet (Excel, Microsoft), and a mathematical formula was to predict expected body composition after weight loss at different weights. The mathematical formula used was based upon typical body composition results from previous weight clinic studies [17, 23]. This enabled an appropriate ideal body weight for each cat to be estimated and this was then used in energy intake calculations. Initial energy allocation was calculated as 35–40 kcal ME (metabolisable energy) x estimated ideal weight (kg), aiming to achieve weight loss at ~ 1% per week [18]. Adjustments to the amount fed were then made based on other factors such as ability to exercise, current energy allocation and owners’ request for gradual rather than sudden acclimatisation to weight loss (e.g. dietary change made over seven days rather than three days). During the initial appointment, lifestyle alterations which would be required to assist in weight loss, such as avoiding extra food or treats, approaches to providing non-food-related rewards and increasing daily activity, were discussed with the owner.

Cats were fed weight management diets formulated to meet essential nutrient requirements when fed for weight loss (Table 5). One diet was wet and two were dry; the wet food and one of the dry foods were high in protein with a medium fibre content (HPMF) whilst the other dry diet was high in both protein and fibre (HPHF). Cats could either be fed dry food exclusively, wet food exclusively, or a mix of wet and dry food, with the choice dependent on the preferences of both owner and cat. Owners weighed all dry food portions with electronic gram scales and, to ensure accuracy of the owners’ scales, a 24 h portion of food was first weighed on calibrated scales (Salter) at the clinic, and then weighed by the client at home.

**Table 5:** Average composition of diets used for weight loss

Criterion	High protein high fibre dry^a^		High protein medium fibre dry^b^		High protein medium fibre wet^c^	
ME content	3070 kcal/kg		3560 kcal/kg		640 kcal/kg	
	Per 100 g AF	g/1000 kcal (ME)	Per 100 g AF	g/1000 kcal (ME)	Per 100 g AF	g/1000 kcal (ME)
Moisture	5.5	18	5.5	15	84	1313
Crude protein	34.0	111	42.0	118	7.5	117
Crude fat	9.0	29	10.0	28	2	31
Digestible carbohydrate^d^	19.1	62	19.8	56	3.1	48
Crude fibre	13.9	45	6.4	18	1.5	23
Total dietary fibre	23.6	77	14.7	41	1.5	23
Ash	8.8	29	8	23	1.9	30

^a^High protein high fibre dry food (Satiety Weight Management, Royal Canin); ^b^High protein medium fibre dry food (Obesity Weight Management, Royal Canin); ^c^High protein medium fibre wet food (Obesity Weight Management, Royal Canin); ^d^Digestible carbohydrate fraction (e.g. sugars and starch) calculated using the following predictive equation: digestible carbohydrate [g] = dry matter [g] - (crude fat [g] + crude protein [g] + ash [g] + total dietary fibre [g]). ME = Metabolizable energy content; AF = as fed; DM = dry matter

All cats were assessed every 14–28 days, depending on the availability of the owner during the weight loss programme. On each occasion body weight measurements were taken, and changes were made to the dietary plan if necessary to maintain steady weight loss. All adjustments to the dietary plan were made by a registered veterinary nurse (SLH). Throughout the weight loss period the owners maintained a diary covering diet ration fed (amount offered and consumed), activity and any additional food that had been consumed. A detailed evaluation was conducted in cats that reached their target weight. These cats were confirmed to have remained healthy based on physical examination, haematology, serum biochemistry and urinalysis (by cystocentesis). Body weight and body condition were recorded, and body composition was assessed by DEXA, aiming for 15–25% body fat after weight loss.

### Classification of outcomes of weight loss

Cats were assigned to three groups depending upon the outcome of their weight loss programme: cats that reached their target weight were classified as ‘completed’; cats that were euthanised prior to reaching their target weight were classified as ‘died’ and the reason for euthanasia was recorded; cats that did not reach target weight for another reason were classified as ‘stopped’, and the reason was recorded where known. This latter category included all cats lost to follow-up because their owners stopped attending the clinic. Before being classified as lost to follow-up, at least three attempts were made to contact the owner by telephone and at least one attempt was made by post.

### Data handling and statistical analysis

Data were recorded in a computer spreadsheet (Additional file 1: Excel For Mac version 15.28, Microsoft Inc.) and statistical analyses were performed with computer software (Stats Direct version 3.0.171; Stats Direct, Altrincham, UK), with the level of significance set at *P* < 0.05 for two-sided analyses. Data are reported as median (range), except where indicated. Sample size was estimated based upon the results from a similar study in dogs [24]. In that study, mean (standard deviation) percentage weight loss was 24.9 ± 8.58% and 10.8 ± 9.27% in dogs that completed and stopped, respectively, and the ratio of dogs that completed to dogs that stopped was approximately 2:1. Assuming an expected power of 90% and alpha of 0.05, it was estimated that at least 33 cats would be needed (22 in the completed and 11 group). However, given that this was a retrospective and observational study, we decided to adopt a similar approach to the previous study in dogs [24], and included all cats that met the eligibility criteria during the study timeframe.

Associations between success with weight loss and various explanatory variables were tested with logistic regression. The outcome variable of interest was success with weight loss, whereby cats that completed their programme reaching target weight were assigned a score of 1, whilst those not completing their programme (either because they had stopped prematurely or had died) were assigned a score of 0. The continuous explanatory variables studied were age, lean body mass, starting body fat percentage, weight loss required, actual weight lost, duration of weight loss, rate of weight loss, metabolisable energy intake during weight loss, the number of times weight loss stalled (i.e. when there was no change in weight or weight gain between appointments), and the number of diet energy intake changes (i.e. when the weight management clinic staff adjusted down the daily food intake at the time of a recheck). The actual weight lost and rate of weight loss were both expressed as a percentage of starting body weight (SBW), whereas rate of weight loss was the average of the whole weight loss period. Duration of weight loss was calculated from the date of the first appointment to the date when target weight was reached (for those completing), or to the last available weight record (for those not completing). Categorical explanatory variables studied included: breed, sex, neuter status, diet characteristics, dietary alterations, and disease status. For breed, since only small numbers of pedigree cats were represented, the proportions of mixed breed and pedigree cats were assessed. The diet used was categorised both by macronutrient content (HPHF vs. HMPF) and by food type (dry food vs. wet food or a mix of wet and dry). Dietary alterations were assessed in three ways: the number of times weight loss stalled (i.e. no weight loss or any weight gain between appointments), the number of times the daily energy intake was reduced, and whether the diet type was switched (e.g. from dry food only to either wet food or a mix if wet and dry). Disease status was characterised as presence or absence of a concurrent disease, presence of more than two concurrent diseases, and the body system affected (e.g. orthopaedic, dermatological, alimentary, cardiovascular, oral, and urinary).

Analyses were performed twice: firstly, cats that completed were compared against all cats that failed to complete (i.e. those that stopped and those that died); subsequently, cats that died were excluded and cats that were completed were compared against those that stopped. Initially, all variables listed above were tested individually with simple logistic regression. A multiple logistic regression model was then built, which initially included the variables identified as *P* < 0.1 on simple regression analysis. Contingency tables were used to assess the independence of each factor compare with all other factors. The model was then refined over multiple rounds using backwards-stepwise elimination, with reference to the Akaike information Criterion (AIC). When removal of any effect yielded a model with lower AIC, the variable with the least effect was removed until the model with the lowest AIC was found, and provided that variables were significant in their own right (*P* < 0.05). Model fit was further assessed using the Pearson chi-square goodness of fit test. Continuous explanatory variables were tested both in continuous and in categorical format (as either tertiles or quartiles as appropriate). In the final model, the choice of competing linear versus categorical formats was based on which model fitted best, with reference to the AIC. Logistic regression results are reported as odds ratios (OR) and 95% confidence intervals (95% CI).

## Additional file

Additional file 1: Computer spreadsheet (Excel, Microsoft; .xlsx) containing study data. (XLSX 16 kb)

## Abbreviations

AF: As fed
BCS: Body condition score
BSH: British shorthair
CI: Confidence intervals
DEXA: Dual-energy X-ray absorptiometry
DSH: Domestic longhair
DSH: Domestic shorthair
F: Female
FS: Female spayed
HPHF: High protein high fibre
HPMF: High protein medium fibre
M: Male
ME: Metabolisable energy
MN: Male neutered
OR: Odds ratio
RCWMC: Royal Canin Weight Management Clinic
